# Assessment of the duration of maternal‐derived antibodies specific to the *Mycoplasma agalactiae* vaccine in goat kids

**DOI:** 10.1002/vms3.888

**Published:** 2022-07-19

**Authors:** Mostafa Abdollahi, Samad Lotfollahzadeh, Taghi Zahraei Salehi, Farhad Moosakhani, Afshin Raoofi

**Affiliations:** ^1^ Faculty of Veterinary Medicine Departmnent of Internal Medicine University of Tehran Tehran Iran; ^2^ Faculty of Veterinary Medicine Department of Microbiology and Immunology University of Tehran Tehran Iran; ^3^ Faculty of Veterinary Medicine Department of Microbiology and Immunology Islamic Azad University Karaj Branch Karaj Iran

**Keywords:** goat kid, immunity, *Mycoplasma agalactiae*, vaccine

## Abstract

**Background:**

Contagious agalactia (CA) is one of the most important diseases in the small ruminant industry in Iran. The historical aetiology of this disease is *Mycoplasma agalactiae* (Ma). The main way to control this disease, in addition to management measures, is vaccination. In ruminant newborns, determining the age of first vaccination against Ma is a challenge due to the interference between colostrum‐derived maternal immunity and vaccination‐induced immunity. The aim of this study was to evaluate the consistency of maternal‐derived antibodies specific to the Ma in goat kids blood serum born from the vaccinated does.

**Objectives:**

Dtermination of level of antibody against Ma in goat kids born from vaccinated dams against Ma. Assessment of duration of protective level of maternal derived antibody in goat kids serum, after receiving colostrum from vaccinted mother with Ma vaccine. Determination the best time vaccination against Ma in goat kids receiving colostrum from vaccinated dams.

**Methods:**

20 Saanen goat kids were studied in two groups of 10 animals including control (receiving colostrum from unvaccinated does) and treatment (receiving colostrum from vaccinated does). Indirect Elisa was used to evaluate serum specific antibodies to Ma in goat kids (control and treatment groups) from birth to 100 days of age.

**Results:**

After receiving a sufficient amount of colostrum, the goat kids in the treatment group had a significantly higher S/P% than the control group until 56 days after birth (*p *< 0.05) and at 70–100 days after birth, there was no significant difference between the treatment and control groups (*p *> 0.05).

**Conclusions:**

This study showed that 56–70 days of age could be a good age to give the first dose of CA vaccine in goat kids, but more studies are needed on the effectiveness of this vaccine at this age.

## Graphical abstract

Maternal‐derived antibodies specific to the Ma in goat kids born from vaccinated does were measured from birth to 100 days old. Results showed that 56–70 days of age is the good age to give the first dose of CA vaccine in goat kids.

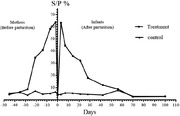

## 1 INTRODUCTION

Contagious agalactia (CA) was first reported in Italy in 1816 as mal di sito (disease of the place), due to disease ability to persist in one place and cause disease in new herds entering the same place (Lambert, 1987). Due to its contagious nature, the disease was named Contagious agalactia in 1871 by the municipality of the Italian metropolitan area (Brusasco) (Madanat et al., 2001). Clinical signs of the CA include mastitis, arthritis, keratoconjunctivitis and occasionally abortion (Thiaucourt & Bolske, 1996; Migliore et al., 2021). The causative agent of the historical aetiology of CA is *Mycoplasma agalactiae* (Ma), which was first isolated and identified in 1923 (Jey & Trady, 2019). In sheep, Ma is still the most important cause of the disease, but in goats the other three pathogens, which include *Mycoplasma mycoides* subspecies *capri* (Mmc) (older name: *Mycoplasma mycoides* LC(, *Mycoplasma putrefaciens* (Mp) and *Mycoplasma capricolum* subspecies *capricolum* (Mcc), can cause CA to a lesser extent (Nicholas et al., 2008; Hajizadeh et al., 2018).

The prevalence of infection to CA causing *Mycoplasmas* in small ruminants in Iran is estimated 20% (Pirali Kheirabadi & Ebrahimi, 2007), 51% (Ashtari et al., 2015), 36% (Shamsaddini Bafti et al., 2017) and 25% (Hajizadeh et al., 2018). Economic losses caused by CA in small ruminant breeding industry can be categorised in three areas including: high morbidity of the disease, reduced production and treatment costs (Jey & Trady, 2019). The main action to control CA in endemic areas and Iranian sheep and goat herds is regular vaccination with the killed vaccine (Hajizadeh et al., 2018; Constable et al., 2016). Prevention of CA in goat kids, which have been born from vaccinated does, depends on receiving enough maternal immunity through colostrum (Constable et al., 2016). Studies showed feeding 200 ml of colostrum immediately after birth and 210 ml/kg in indoor situation and 280 ml/kg in outdoor raising, during first 24 h after birth would be enough for receiving good passive immunity in goat kids (Matthews, 2016). To date, no studies have been performed on the appropriate age for the first vaccination against CA in goat kids, and the only suggestion is early vaccination of these newborns after the age of 70 days (Constable et al., 2016). There are currently two types of vaccines against Ma in the Iranian market. One is the monovalent vaccine: Agalactia Vac (killed vaccine inactivated by formaldehyde, contains saponin adjuvant, for all age groups of animals, Razi Institute, Alborz, Iran) and the other is the polyvalent vaccine: Agalaksivac (killed vaccine inactivated by phenol, contains Montanid ISA 206 adjuvant, for all age groups of animals, Vetal Company, Gaziantep, Turkey). Studies in Iran have shown that the main cause of CA in goats is Ma, but Mcc, Mp and Mmc are also among the etiological agents of CA in goats (Shamsaddini Bafti et al., 2017; Hajizadeh et al., 2018). Therefore, in the Iranian goat breeding industry, the use of the polyvalent vaccine (Agalaksivac) seems to be a better choice.

Due to some high‐risk diseases of small ruminants in Iran such as foot and mouth disease (FMD) (Nikvand et al., 2019), Peste des petits ruminants (PPR) (Bazarghani et al., 2006), sheep pox (Mirzaie et al., 2015), goat pox (Mirzaie et al., 2015), Contagious ecthyma (Davari et al., 2015), Enterotoxemia (Ahmadi Rahnemoon et al., 2020), Pasteurellosis (Valadan et al., 2014), CA (Hajizadeh et al., 2018), there is a heavy traffic of newborn vaccines in the sheep and goat herd health program up to the age of 100 days. A proper order based on scientific findings in this heavy traffic is needed to achieve a better health index in small ruminants herds.

The aim of this study was to evaluate the duration of maternal immunity against Ma in goat kids after receiving colostrum from mother vaccinated with Agalaksivac oil vaccine, from birth to 100 days of age.

## 2 MATERIALS AND METHODS

### 2.1 Study area

This study was conducted for one year (September 2020 to September 2021) in a farm with 1500 Saanen goats (Ajdad Sepidan Kosar located in Qarchak city, Tehran Province, Iran). There was no history of CA on this farm. Immunization of all animals against CA was performed on the farm by vaccination with Agalaksivac vaccine (Vetal Company, Gaziantep, Turkey) every 6 months. The sample size was determined based on previous studies (Sotoodehnia et al., 2005; Ozdemir et al., 2019; Elfil & Negida, 2017).

### 2.2 Animals

Twenty seronegative Saanen female goats, aged 10 months, were included in study. Indirect Elisa test (Ma indirect Elisa commercial kit, MAGALS‐5SP, ID‐Vet, Grabels, French) was used for detection of antibody against Ma in blood serum of chosen animals. Animals were allocated to two groups of 10 heads. Animals in one group received Agalaksivac (killed vaccine, Vetal Company, Gaziantep, Turkey) subcutaneously (treatment group) and in the other group including 10 goats 0.9% sodium chloride was injected as placebo (control group). The vaccine and placebo were injected under the skin of the neck. Singleton pregnancy of the study animals was confirmed by ultrasound (Buckrell, 1988). Grouping was done by simple random method (lottery method) (Elfil & Negida, 2017).

#### 2.2.1 Newborn kids

After birth, kids with appropriate weight range (2.5–3.5 kg) (Ince, 2010) were included in the study and fed with enough colostrum (200 ml immediately after birth and 210 ml/kg in the first 24 h) (Matthews, 2016). Colostrum was prepared from the mother of each newborn kids, without measuring IgG. To determine the absence of failure of passive transfer (FPT) in kids, a blood sample was taken from each one 48 h after birth and serum total IgG concentration was measured using Elisa method (DEIA640, CD Creative Diagnostics, Shirley, USA) (Batmaz et al., 2019). In this study, all chosen does had singleton pregnancy and have given birth to one kid, then 20 newborn kids participated in two groups of 10 heads. Infants born to mothers receiving the vaccine were in the treatment group and infants born to mothers receiving the placebo were in the control group.

### 2.3 Vaccination

Due to the permissibility of using the vaccine in the second half of pregnancy in the vaccine manufacturer's instructions, the animals received a dose of vaccine or placebo on the 105th day of pregnancy according to the group and this administration was repeated on the 120th day of pregnancy (Constable et al., 2016). For vaccination, the polyvalent Agalaksivac (killed vaccine, Vetal Company, Gaziantep, Turkey) with three strains including Ma, Mcc and *Mycoplasma mycoides* subsp. *mycoides* (Mmm) was used. This vaccine is oily in nature, inactivated by phenol and contains Montanid ISA 206 adjuvant. Each dose (2 ml) of the vaccine contains 1–2 × 10^9^ of each of the three organisms. The exact concentration of these microorganisms per millilitre of vaccine is not available.

### 2.4 Serum collection

In does, blood samples were taken on the 105th day of pregnancy (45 days before parturition) and continued weekly until parturition (day 150 of pregnancy) (Bodjo et al., 2006). In newborn kids, the first blood sample was taken immediately after birth (15 min after birth and 10 min before eating the first meal of colostrum) and then on days 3, 7, 14, 21, 28, 42, 56, 70 and 100 after birth (Bodjo et al., 2006; Veschi et al., 2006).

### 2.5 Entry and exit criteria

Inclusion criteria were considered for mothers (complete clinical health, singleton pregnancy, having a good body condition score [3 on the 5‐point scale]) and for newborn kids (complete clinical health, delivery without intervention, appropriate weight) (Vries et al., 2014). Exclusion criteria were considered for mothers (disease incidence) and for infants (failure of passive transfer, disease incidence) (Vries et al., 2014).

### 2.6 Serological analysis

Ma indirect Elisa commercial kit (MAGALS‐5SP, ID‐Vet, Grabels, French) was used to determine the presence of Ma antibody in serum of all studied animals. Goat IgG Sandwich Elisa commercial kit (DEIA640, CD Creative Diagnostics, Shirley, USA) was used to determine the serum total IgG concentration in all studied newborn kids. These tests were performed according to the manufacturer's instructions, by an expert in the Laboratory of Immunology, Department of Microbiology and immunology, University of Tehran, Iran.

### 2.7 Statistical analysis

The normal distribution of data in each group was confirmed using Shapiro–Wilk test. After plotting the S/P%–time model in GraphPad Prism software (Version 7) for S/P% obtained in indirect Elisa, independent *T*‐test in SPSS software (Version 22) was used for statistical analysis of data. Statistically significant differences were declared at a *p* value of less than 0.05.

## 3 RESULTS

**FIGURE 1:**
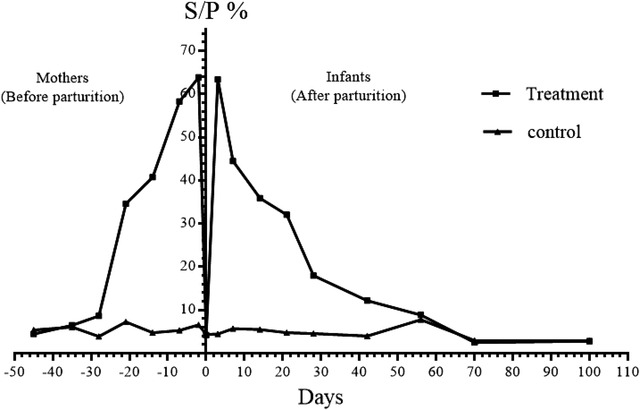
S/P%–time diagram in mothers and newborn kids participating in the study

No clinical signs of any disease were observed during the study in does and newborn kids. FPT did not occur in any of the neonates studied. In mothers, as shown in Table 1 and Figure 1, on days 45 and 35 before parturition, there was no significant difference in Elisa S/P% between the control and treatment groups (*p *> 0.05). On 28, 21, 14, 7 and 2 days before parturition, the Elisa S/P% were significantly higher in the treatment group as compared with S/P% in the control group (*p *< 0.05).

**TABLE 1:** S/P% in mothers participating in the study (mean ± SD)

Day (before parturition)	Control group	Treatment group	*p* Value	Interpretation
–45	5.4 ± 1.9	4.4 ± 1.4	=0.191	*μ_C_* = *μ_T_*
–35	6.1 ± 1.2	6.6 ± 4.5	=0.738	*μ_C_* = *μ_T_*
–28	3.9 ± 0.8	8.7 ± 2.5	≤0.001	*μ_C_* < *μ_T_*
–21	7.3 ± 2.0	34.6 ± 2.8	≤0.001	*μ_C_* < *μ_T_*
–14	4.8 ± 0.9	40.8 ± 7	≤0.001	*μ_C_* < *μ_T_*
–7	5.3 ± 1.6	58.3 ± 8.5	≤0.001	*μ_C_* < *μ_T_*
–2	6.6 ± 0.6	63.9 ± 3.3	≤0.001	*μ_C_* < *μ_T_*

*μ*: mean, *C*: control group, *T*: treatment group.

In newborn kids, as shown in Table 2 and Figure 1, before receiving colostrum, the Elisa S/P% were not significantly different between the control and treatment groups (*p *> 0.05). On days 3, 7, 14, 21, 28, 42 and 56 after birth, the Elisa S/P% in kids in the treatment group were significantly higher than kids in the control group (*p *< 0.05). At 70 and 100 days after birth, there was no significant difference in Elisa S/P% between the kids in the control and treatment groups (*p *> 0.05).

**TABLE 2:** S/P% in kids participating in the study (mean ± SD)

Day (after birth)	Control group	Treatment group	*p* Value	Interpretation
0	4.4 ± 1.3	4.2 ± 0.9	=0.694	*μ_C_* = *μ_T_*
3	4.5 ± 0.5	63.4 ± 3.0	≤0.001	*μ_C_* < *μ_T_*
7	5.7 ± 2.0	44.5 ± 6.8	≤0.001	*μ_C_* < *μ_T_*
14	5.5 ± 2.1	35.9 ± 4.2	≤0.001	*μ_C_* < *μ_T_*
21	4.8 ± 1.4	32.1 ± 6.4	≤0.001	*μ_C_* < *μ_T_*
28	4.6 ± 0.4	18.0 ± 1.1	≤0.001	*μ_C_* < *μ_T_*
42	4.0 ± 1.3	12.2 ± 4.1	≤0.001	*μ_C_* < *μ_T_*
56	3.8 ± 0.8	8.9 ± 1.2	≤0.001	*μ_C_* < *μ_T_*
70	3.0 ± 0.7	2.5 ± 1.5	=0.352	*μ_C_* = *μ_T_*
100	2.9 ± 0.6	2.8 ± 0.6	=0.714	*μ_C_* = *μ_T_*

*μ*: mean, *C*: control group, *T*: treatment group.

## 4 DISCUSSION

CA is one of the most important diseases in the small ruminant breeding industry in Iran (Pirali Kheirabadi & Ebrahimi, 2007; Ashtari et al., 2015). During a study by Pirali Kheirabadi and Ebrahimi (2007), 101 healthy sheep were studied in the west of Iran. The samples included milk and conjunctival swab. PCR test was used. Twenty per cent of the studied animals were positive for Ma infection. During this study, it was found that in the west of Iran, 77% of sheep herds have at least one ewes infected by Ma (Pirali Kheirabadi & Ebrahimi, 2007). In a study by Ashtari et al. (2015), 91 sheep (suspected CA) were studied in Khuzestan province of Iran. The samples included milk, conjunctival swab, ear swab and joint fluid. PCR test and *Mycoplasma* culture were used. In *Mycoplasma* culture method, 37% of the samples were positive. In PCR test, 51.65% of the samples were positive for *Mycoplasma*, of which 8.79% were related to Ma. During this study, it was found that the conjunctival swab is the best sample for identification of *Mycoplasma* and other etiological agents are involved in the occurrence of CA, in Khuzestan province of Iran (Ashtari et al., 2015). In a study by Shamsaddini Bafti et al. (2017), 132 sheep and goats (suspected CA) were studied in the southeast of Iran. The samples included milk, conjunctival swab, ear swab and joint fluid. PCR test and *Mycoplasma* culture were used. In *Mycoplasma* culture method, 14.8% of the samples were positive. In PCR test, 36% of the samples were positive for *Mycoplasma*, of which 6.1% were related to Ma. During this study, it was found that in the southeast of Iran, other etiological agents are involved in the occurrence of CA, which are unknown (Shamsaddini Bafti et al., 2017). In a study by Hajizadeh et al. (2018), 132 sheep and goats (suspected CA) were studied in northwest Iran. The samples included milk, conjunctival swab and joint fluid. PCR test was used. Twenty‐five per cent of the studied animals were positive for *Mycoplasma* infection. Among infected animals, 54% had infection with more than one *Mycoplasma* species. Among the infected animals, 47.2% had Ma infection, 43.4% had Mp infection, 7.5% had Mcc infection and 1.9% had Mmc infection. During this study, for the first time in Iran, Mp and Mmc were isolated from small ruminant flocks (Hajizadeh et al., 2018).

According to the instructions of the used Elisa kit in this study, S/P% ≥ 60% is equal to the natural infection with Ma. Agalaksivac vaccine caused the S/P% ≥60% in all vaccinated female goats (63.9± 3.3 two days before parturition) as well as goat kids (63.4± 3.0 three days after birth) that were fed enough colostrum of vaccinated does (200 ml immediately after birth and 210 ml/kg in the first 24 h) (Figure 1). On day 70 after birth, there was no difference in maternal immunity levels against Ma among goat kids born to vaccinated and non‐vaccinated mothers. According to the results, the persistence of maternal immunity against Ma ends at the age of 56–72 days after birth.

During the first one‐third of pregnancy, primary lymphoid tissue (bone marrow and thymus) forms in the fetus, and B and T cells become apparent in the foetal blood. During the middle and final one‐third of pregnancy, secondary lymphoid tissues development occurs, such as MALT (mucosa‐associated lymphoid tissue) (Marchant & Kollmann, 2015). At this time, the fetus is able to produce an immune response to a range of pathogens. The immune system in animals develops significantly during the first months of life and continues until the animal matures (Dowling & Levy, 2014). Although not determined in goat kids, in calves, the immune system matures at 6 months of age (Chase et al., 2008).

In ruminants, the placenta is syndesmochorial. It is not possible to transmit antibodies throughout the blood from mother to infant, and the maternal immunity is transmitted to infants only through colostrum feeding (Hernandez‐Castellano et al., 2015). One of the important principles in the livestock breeding industry is the reception of colostrum by the newborn at a limited time after birth (up to 24 h after birth) (Matthews, 2016). Although maternal immunity is important for the survival of the infants, this immunity is like a double‐edged sword because it inhibits the humoral endogenous immune response (such as the vaccine response) until it is sufficiently degraded (Day & Schultz, 2014).

Due to the delayed maturation of the immune system in newborns, vaccination at an early age may be less effective than in adult animals (Bodjo et al., 2006). In this study, we found that suitable time for vaccination against Ma in goat kids which received enough amount of colostrum from vaccinated does is 56–70 days after birth. Vaccines are an important tool for the prevention of CA (Constable et al., 2016). To date, several vaccines have been developed for the CA, and there is still no single vaccine for the disease universally (Nicholas et al., 2008).

To date, few attempts have been made to develop a preventive vaccination for infection with Mmc and Mp (Nicholas et al., 2008). Although the disease caused by these pathogens can be severe, their prevalence is relatively low (Bajmocy et al., 2000). Studies have been performed to identify the immunogenic proteins of *Mycoplasmas* that cause CA. In addition, advances in epitope mapping have facilitated the identification of immunogenic proteins that can be used to make subunit vaccines (Nicholas et al., 2008). A strongly immunogenic surface lipoprotein (AvgC) has been identified in Ma that is exposed to the surface and stimulates the production of specific antibodies in sheep serum (Santona et al., 2002). In *Mycoplasma capricolum subsp. capripneumoniae* (Mccp), 20 immunogenic proteins have been identified in the whole cell, 9 of which are located in the cell membrane. Membrane proteins appear to be primarily immunogenic proteins for Mccp (Zhao et al., 2012). In Mmc, 20 immunogenic proteins have been identified in the whole cell (Churchward et al., 2015). These proteins may have good potential for making subunit vaccines.

## 5 CONCLUSION

Based on the results obtained and discussion, it can be said, if enough colostrum (21% of body weight) is fed to goat kids at the appropriate time (the first 24 h of life), vaccination of goat kids against Ma at the age of 56–70 days after birth can be a good option in goat herds. During the search of authors in scientific references, no other studies were found on the stability of maternal immunity against *Mycoplasmas* in goat kids, and it seems that this is the first study on the stability of maternal immunity against Ma in goat kids and determination of right time for vaccination against Ma in goat kids.

## CONFLICT OF INTEREST

The authors declare that there is no conflict of interest.

## AUTHOR CONTRIBUTIONS

Mostafa Abdollahi: Conception, performing the experiment, analysing the data, writing the article. Samad Lotfollahzadeh: Conception and design, collecting the data, writing and revising the article. Taghi Zahraei Salehi: Conception. Farhad Moosakhani: Conceived the experiment, assistance in laboratory work. Afshin Raoofi: Study advice.

## FUNDING

There is no source of fund to declare.

## ETHICAL APPROVAL

The authors confirm that the ethical policies of the journal, as noted on the journal's author guidelines page, have been adhered to and the appropriate ethical review committee approval has been received.

## ETHICAL CONSIDERATION

Before starting the study, the proposal was sent to the research committee of Ajdad Sepidan Kosar Company and the code of ethics in the research was taken (ethics code: 1400/471).

### PEER REVIEW

The peer review history for this article is available at https://publons.com/publon/10.1002/vms3.888

## Data Availability

The data that support the findings of this study are available within the manuscript and also are available from the corresponding author upon reasonable request.

## References

[vms3888-bib-0001] Ahmadi Rahnemoon, A. , Esmaeilzadeh, S. , Mohammadian, B. , Ghorbanpoor, M. , & Ghadrdan Mashhadi, A. (2020). Ovine enterotoxemia in Ahvaz region: Pathological, bacteriological, serological and molecular studies. Iranian Veterinary Journal, 16, 25–39.

[vms3888-bib-0002] Ashtari, A. , Pourbakhsh, S. A. , Ghaemmaghami, S. , Looni, R. , Pooladgar, A. R. , & Ali Shirudi, A. (2015). Isolation and identification of *Mycoplasma agalactiae* by culture and polymerase chain reaction (PCR) from affected sheep to Contagious agalactia of Khuzestan province, Iran. Archives of Razi Institute, 70, 21–27.

[vms3888-bib-0003] Bajmocy, E. , Turcsanyi, I. , Bolske, G. , Bacsadi, A. , & Kiss, I. (2000). Disease caused by *Mycoplasma mycoides subspecies mycoides LC* in Hungarian goat herds. Acta Veterinaria Hungarica, 48, 277–283. 11402711 10.1556/AVet.48.2000.3.4

[vms3888-bib-0004] Batmaz, H. , Kacar, Y. , Topal, O. , Mecitoglu, Z. , Gumussoy, K. S. , & Kaya, F. (2019). Evaluation of passive transfer in goat kids with Brix refractometer and comparison with other semiquantitative tests. Turkish Journal of Veterinary and Animal Sciences, 43, 596–602.

[vms3888-bib-0005] Bazarghani, T. T. , Charkhkar, S. , Doroudi, J. , & Bani Hassan, E. (2006). A review on peste des petits ruminants (PPR) with special reference to PPR in Iran. Journal of Veterinary Medicine, 53, 17–18.

[vms3888-bib-0006] Bodjo, S. C. , Couacy‐Hymann, E. , Koffi, M. Y. , & Danho, T. (2006). Assessment of the duration of maternal antibodies specific to the homologous peste des petits ruminant vaccine “Nigeria 75/1" in Djallonké lambs. Biokemistri, 18, 99–103.

[vms3888-bib-0007] Buckrell, B. C. (1988). Applications of ultrasonography in reproduction in sheep and goats. Theriogenology, 29, 71–84.

[vms3888-bib-0008] Chase, C. C. L. , Hurley, D. J. , & Reber, A. J. (2008). Neonatal immune development in the calf and its impact on vaccine response. Veterinary Clinics of North America: Food Animal Practice, 24, 87–104. 18299033 10.1016/j.cvfa.2007.11.001 PMC7127081

[vms3888-bib-0009] Churchward, C. P. , Rosales, R. S. , Gielbert, A. , Dominguez, M. , Nicholas, R. A. J. , & Ayling, R. D. (2015). Immunoproteomic characterisation of *Mycoplasma mycoides* subspecies *capri* by mass spectrometry analysis of two‐dimensional electrophoresis spots and western blot. Journal of Pharmacy and Pharmacology, 67, 364–371. 25495903 10.1111/jphp.12344

[vms3888-bib-0010] Constable, P. D. , Hinchcliff, K. W. , Done, S. H. , & Grunberg, W. (2016). Diseases of the mammary gland in veterinary medicine (11th ed., pp. 1994–1996). Philadelphia: Saunders Elsevier.

[vms3888-bib-0011] Davari, S. A. , Sayyari, M. , & Mohammadi, A. (2015). Histopathological study and F1L gene sequence analysis of contagious ecthyma in small ruminants of Shiraz suburb, Iran. Tropical Biomedicine, 32, 335–343. 26691262

[vms3888-bib-0012] Day, M. J. , & Schultz, R. D. (2014). Immune system ontogeny and neonatal immunology in veterinary immunology – Principles and practice (2nd ed., pp. 213–220). Bristol, UK: CRC Press.

[vms3888-bib-0013] Dowling, D. J. , & Levy, O. (2014). Ontogeny of early life immunity. Trends in Immunology, 35, 299–310. 24880460 10.1016/j.it.2014.04.007 PMC4109609

[vms3888-bib-0014] Elfil, M. , & Negida, A. (2017). Sampling methods in clinical research: An educational review. Emergency, 5, 52–55. PMC5325924 28286859

[vms3888-bib-0015] Hajizadeh, A. , Ghaderi, R. , & Ayling, D. D. (2018). Species of *Mycoplasma* causing Contagious agalactia in small ruminants in northwest Iran. Veterinaria Italiana, 54, 205–210. 30574997 10.12834/VetIt.831.4072.2

[vms3888-bib-0016] Hernandez‐Castellano, L. E. , Morales‐delaNuez, A. , Sanches‐Macias, D. , Moreno‐Indias, I. , Torres, A. , Capote, J. , Arguello, A. , & Castro, N. (2015). The effect of colostrum source (goat vs. sheep) and timing of the first colostrum feeding (2h vs. 14h after birth) on body weight and immune status of artificially reared newborn lambs. Journal of Dairy Science, 96, 204–210. 10.3168/jds.2014-8350 25468691

[vms3888-bib-0017] Ince, D. (2010). Reproduction performance of Saanen goats raised under extensive conditions. African Journal of Biotechnology, 9, 8253–8256.

[vms3888-bib-0018] Jay, M. , & Tardy, F. (2019). Contagious agalactia in sheep and goats: Current perspectives. Veterinary Medicine (Auckland), 10, 229–247. 10.2147/VMRR.S201847 PMC6938181 31921613

[vms3888-bib-0019] Lambert, M. (1987). Contagious agalactia of sheep and goats. Revue Scientifique et Technique, 6, 681–711. 32370341 10.20506/rst.6.3.308

[vms3888-bib-0020] Madanat, A. , Zendulkova, D. , & Pospísil, Z. (2001). Contagious agalactia of sheep and goats: A review. Acta Veterinaria Brno, 70, 403–412.

[vms3888-bib-0021] Marchant, A. , & Kollmann, T. R. (2015). Understanding the ontogeny of the immune system to promote immune‐mediated health for life. Frontiers in Immunology, 6, 1–3. 25755655 10.3389/fimmu.2015.00077 PMC4337332

[vms3888-bib-0022] Matthews, J. (2016). Weak kids in diseases of the goat (4th ed., pp. 69–79). New York, US: Wiley Blackwell.

[vms3888-bib-0023] Migliore, S. , Puleio, R. , Nicholas, R. A. , & Loria, G. R. (2021). *Mycoplasma agalactiae*: The sole cause of classical Contagious agalactia? Animals, 11, 1782. 10.3390/ani11061782 PMC8232315 34203625

[vms3888-bib-0024] Mirzaie, K. , Barani, S. M. , & Bokaie, S. (2015). A review of sheep pox and goat pox: Perspective of their control and eradication in Iran. Journal of Advanced Veterinary and Animal Research, 2, 373–381.

[vms3888-bib-0025] Nicholas, R. A. J. , Ayling, R. D. , & McAuliffe, L. (2008). Contagious agalactia in mycoplasma diseases of ruminants (1st ed., pp. 98–113). Surrey, UK: CAB International.

[vms3888-bib-0026] Nikvand, A. A. , Jalali, S. M. , Ghadrdan, M. A. , Nori, M. , & Hassanpoor, A. S. (2019). Survey on thyroid hormones and their relationship with serum biomarkers of myocardial injury in cattle, sheep and lambs with FMD. Scientific Research Iranian Veterinary Journal, 15, 92–101.

[vms3888-bib-0027] Ozdemir, U. , Ali Turkyılmaz, M. , & Nicholas, R. A. J. (2019). A live vaccine for Contagious agalactia is protective but does not provoke an ELISA response. Animal Husbandry, Dairy and Veterinary Science, 3, 1–3.

[vms3888-bib-0028] Pirali Kheirabadi, K. H. , & Ebrahimi, A. (2007). Investigation of *Mycoplasma agalactiae* in milk and conjunctival swab samples from sheep flocks in west cental, Iran. *Pakistan Journal of Biological Sciences*, 10, 1346–1348. 10.3923/pjbs.2007.1346.1348 19069942

[vms3888-bib-0029] Santona, A. , Carta, F. , Fraghi, P. , & Turrini, F. (2002). Mapping antigenic sites of an immunodominant surface lipoprotein of *Mycoplasma agalactiae*, AvgC, with the use of synthetic peptides. Infection and Immunity, 70, 171–176. 11748179 10.1128/IAI.70.1.171-176.2002 PMC127643

[vms3888-bib-0030] Shamsaddini Bafti, M. , Pourbakhsh, S. A. , Ezatkhah, M. , & Ashtari, A. (2017). Detection of *Mycoplasma agalactiae* in small ruminants of Southeast Iran. Archives of Razi Institute, 72, 237–242. 30315700 10.22092/ari.2017.113302

[vms3888-bib-0031] Sotoodehnia, A. , Moazeni Jula, G. , Madani, R. , Golchinfar, F. , & Naserirad, A. (2005). Determination of antibody response against inactivated agalactia vaccine in small ruminants. Archives of Razi Institute, 60, 67–75.

[vms3888-bib-0032] Thiaucourt, F. , & Bolske, G. (1996). Contagious caprine pleuropneumonia and other pulmonary mycoplasmoses of sheep and goats. Revue Scientifique et Technique, 15, 1397–1414. 9190020 10.20506/rst.15.4.990

[vms3888-bib-0033] Valadan, M. , Jabbari, A. R. , Niroumand, M. , Tahamtan, Y. , & Bani Hashemi, S. R. (2014). Isolation and identification of *Pasteurella multocida* from sheep, & goat in Iran. Archives of Razi Institute, 69, 47–55.

[vms3888-bib-0034] Veschi, J. L. A. , Dutra, I. S. , Miyakawa, M. E. F. , Perri, S. H. V. , & Uzal, F. A. (2006). Immunoprophylactic strategies against enterotoxemia caused by Clostridium perfringens type D in goats. Pesquisa Veterinaria Brasileira, 26, 51–54.

[vms3888-bib-0035] Vries, R. B. M. D. , Wever, K. E. , Avey, M. T. , Stephens, M. L. , Sena, E. S. , & Leenaars, M. (2014). The usefulness of systematic reviews of animal experiments for the design of preclinical and clinical studies. ILAR Journal, 55, 427–437. 25541545 10.1093/ilar/ilu043 PMC4276599

[vms3888-bib-0036] Zhao, P. , He, Y. , Chu, Y. , Gao, P. , Zhang, X. , Zhang, N. , Zhao, H. , Zhang, K. , & Lu, Z. (2012). Identification of novel immunogenic proteins in *Mycoplasma capricolum subsp. capripneumoniae* strain M1601. The Journal of Veterinary Medical Science, 74, 1109–1115. 22673397 10.1292/jvms.12-0095

